# Infectious complications and NK cell depletion following daratumumab treatment of Multiple Myeloma

**DOI:** 10.1371/journal.pone.0211927

**Published:** 2019-02-13

**Authors:** Hareth Nahi, Michael Chrobok, Charlotte Gran, Johan Lund, Astrid Gruber, Gösta Gahrton, Per Ljungman, Arnika Kathleen Wagner, Evren Alici

**Affiliations:** 1 Division of Hematology, Department of Medicine Huddinge, Karolinska Institutet, Stockholm, Sweden; 2 Department of Hematology, Karolinska University Hospital, Stockholm, Sweden; 3 Department of Clinical Chemistry, Karolinska University Hospital, Stockholm, Sweden; 4 Department of Cellular Therapy and Allogeneic Stem Cell Transplantation, Karolinska University Hospital, Stockholm, Sweden; 5 Cell Therapies Institute, College of Allopathic Medicine, Nova Southeastern University, Fort Lauderdale, Florida; Rush University, UNITED STATES

## Abstract

Treatment with Daratumumab (Dara), a monoclonal anti-CD38 antibody of IgG1 subtype, is effective in patients with multiple myeloma (MM). However, Dara also impairs the cellular immunity, which in turn may lead to higher susceptibility to infections. The exact link between immune impairment and infectious complications is unclear. In this study, we report that nine out of 23 patients (39%) with progressive MM had infectious complications after Dara treatment. Five of these patients had viral infections, two developed with bacterial infections and two with both bacterial and viral infections. Two of the viral infections were exogenous, i.e. acute respiratory syncytial virus (RSV) and human metapneumovirus (hMPV), while five consisted of reactivations, i.e. one herpes simplex (HSV), 1 varicella-zoster (VZV) and three cytomegalovirus (CMV). Infections were solely seen in patients with partial response or worse. Assessment of circulating lymphocytes indicated a selective depletion of NK cells and viral reactivation after Dara treatment, however this finding does not exclude the multiple components of viral immune-surveillance that may get disabled during this monoclonal treatment in this patient cohort. These results suggest that the use of antiviral and antibacterial prophylaxis and screening of the patients should be considered.

## Introduction

During past decades, due to the increase in treatment options, the survival rate of patients with multiple myeloma (MM) has increased dramatically. With the recent introduction of monoclonal antibodies, such as Daratumumab (Dara) to treat MM, immunotherapy has rapidly become indispensable in the management of the disease. Dara was approved in 2015 by the U.S. Food and Drug Administration (FDA) for patients who had at least three prior lines of treatment including one proteasome inhibitor (PI) and one immunomodulatory imide drug (IMiD) or who were refractory to both.

Dara is a human anti-CD38 antibody of IgG1 isotype. The mechanisms of action of Dara include Fc-dependent complement-dependent cytotoxicity (CDC) and antibody-dependent cellular phagocytosis (ADCP), but most of the effect is ascribed to antibody-dependent cellular cytotoxicity (ADCC), which is primarily mediated by NK cells and to some extent also by macrophages [1].

Daratumumab treatment as single-agent in patients with MM shows promising results in 30% of the patients [2]. To date, it is the only single-agent treatment, which shows this rapid decrease of M-component [2]. Dara has also demonstrated superior efficacy in combination with other approved medications for MM, including lenalidomide, dexamethasone and bortezomib [3–5]. However, lymphocyte counts drop after Dara infusion, likely due to their expression of CD38 [6,7]. Therefore, these patients are theoretically at risk for infectious complications [2,8,9]. The precise nature of these defects on cellular immunity are currently unknown.

Infections are one of the leading causes of morbidity and mortality of MM patients. MM patients have a seven-fold increased risk of infectious complications where viral infections are 10-fold more common, and herpes zoster infections are dominating in the list of comorbidities in this patient group with a 14.8-fold increased risk [10]. Inherent immune defects related to the primary disease process, such as reduced NK cell counts and impaired NK cell activity, as well as therapy-related changes of the immune status, may lead to multifactorial pathogenesis of infections. Specifically, declining numbers of CD38-expressing NK cells and subsets of T cells combined with a reported oligoclonality of both CD4^+^ and CD8^+^ T cells leads to an ineffective antiviral innate and adaptive immunity [11]. Novel therapies and the resulting prolonged survival of MM patients have enabled clinicians to observe that tumor progression correlates negatively with immunocompetence of the individual. Furthermore, cumulative therapies of IMiDs and PIs in relapse and refractory MM have resulted in an increased incidence of infections compared to conventional therapies [12]. The reasons for increased infections remain unknown for IMIDs while a characteristic spectrum of infections has been described for other treatment agents. A transient and partially reversible immunosuppressive effect has been shown for PIs, which in turn may increase the prevalence of viral reactivations. Notably, bortezomib treatment can lead to a 4-fold increase incidence of varicella-zoster virus (VZV) reactivation compared to dexamethasone [13]. In addition, cytomegalovirus (CMV) reactivation in MM patients undergoing treatments has been reported to range between 7% to 20% [14,15]. Elotuzumab, a monoclonal anti-SLAMF7 antibody, that has no single agent effect but is used in combination with IMIDs, PIs and steroids, also leads to an increased incidence of herpes zoster compared to both IMIDs and steroid treatments [16].

In this study, we have analyzed the pattern of infectious complications in 23 MM patients that were heavily pretreated before initiation of Dara as a single agent. All patients had undergone multiple prior treatments with PIs, IMiDs, and autologous or allogeneic stem cell transplantations (Allo-SCTs) and did not have any alternative treatments available. Due to the condition of the patients included in this study and the novelty of the treatment, all patients, therefore, were meticulously monitored for safety, efficacy and the occurrence of infectious complications.

## Methods

### Patients

Twenty-three patients were included in compassionate use programs per disease criteria in previously described phase II studies except for the exclusion of patients with creatinine clearance <20ml/min [2,8]. All 23 patients with progressing MM required systemic therapy and were heavily pretreated with multiple lines of treatment including but not limited to IMiDs, PIs, chemotherapy, autologous as well as allogeneic stem cell transplantation. All patients had an Eastern Cooperative Oncology Group (ECOG) performance status of 2 or lower and a measurable M-protein and/or free light chains (FLCs) per International Myeloma Working Group (IMWG) guidelines [17]. Treatment responses were assessed per IWMG guidelines. Patient characteristics are described in Table 1.

**Table 1:** Patient characteristics.

Characteristics of patients that received Daratumumab treatment	
Variable	Number of patients (%)
Total number of patients	23 (100)
Age, mean (range)	63 (34–82)
Gender: female	6 (29)
Myeloma isotype	
IgG	12 (52)
IgA	3 (13)
No M-protein, BJ	8 (35)
eGFR Cockcroft-Gault	85 (14–177)
30–59 (CKD stage 3)	2 (8%)
15–29 (CKD stage 4)	1 (4%)
<15 (CKD stage 5)	1 (4%)
Bone lesion/s, n (%)	22 (96)
**Laboratory values**	
Creatinine (mmol/L), mean (range)	80 (36–567)
Ca (mmol/L), mean (range)	2.28 (1.98–2.92)
Hb (g/L), mean(range)	103 (85–141)
Albumin (g/L), mean(range)	33 (16–39)
Prior lines of therapy, median (range)	3 (2–6)
Prior treatment	
PIs	23 (100)
Bortezomib	23 (100)
Carfilzomib	3 (13)
IMiDs	23 (100)
Lenalidomide	23 (100)
Pomalidomide	12 (52)
Thalidomide	10 (43)
HDT	12 (52)
ASCT	2 (10)

Patients received 9 (2–34) infusions of Dara. All patients had exhausted all available treatment options and had advanced MM with resistance to previously used anti-myeloma drugs. The median inclusion age was 63 (34–82) years. CKD = chronic kidney disease, HDT = high dose treatment; ASCT = allogeneic stem cell transplantation.

The estimated glomerular filtration rates (eGFRs) were calculated using the modification of diet in renal disease (MDRD) formula as described earlier [18]. Renal function was classified according to U.S. national kidney foundation kidney disease outcomes quality initiative (KDOQI) chronic kidney disease (CKD) classification: stage [19].

### Study design and overview

The study was approved by the Stockholm regional ethics committee (Dnr 2014/526-31/3 and 2015/973-32) and was conducted according to the principles of the Declaration of Helsinki, the International Conference on Harmonization, and the Guidelines for Good Clinical Practice. Written informed consent was obtained from all patients in the study. The study was conducted as a cohort study of patients with exhausted treatment options that were included in two separate compassionate of use programs initially offered by Genmab A/S (Copenhagen, Denmark) and thereafter by Janssen Biotech (Solna, Sweden). None of the patients was participating in other studies at the time of inclusion.

### Daratumumab administration and blood sampling

Dara infusions were scheduled according to the recommendation of weekly infusion of 16mg/kg for eight weeks followed by infusion every two weeks for sixteen weeks and thereafter infusions every four weeks. All 23 patients received Dara treatment until progression of MM as defined by IMWG criteria. The treatment was only interrupted during infectious complication. Patients received premedication in the form of corticosteroids, antihistamines and antipyretic before each infusion of Daratumumab.

Blood was collected in conjunction with Dara infusion. Samples were drawn in EDTA collection tubes, (BD Vacutainer EDTA Becton Dickinson and Company (BD) Vacutainer, NJ, USA) 1–2 minutes before the start of each Dara infusion for all patients.

### Detailed analysis of circulating cell populations by flow cytometry

The impact of Dara treatment on circulating lymphocytes was assessed during the peri-treatment period. Briefly, cells were stained for flow cytometric analyses of NK cells and T cell populations, as described previously [7]. Briefly, vitally frozen peripheral blood mononuclear cells (PBMCs) from all patients were thawed, washed and resuspended in cold phosphate-buffered saline (PBS) supplemented with 2% fetal bovine serum (FBS) and 1mM Ethylenediaminetetraacetic acid (EDTA). All antibody stainings for flow cytometry were performed by incubating the cells with previously titrated and validated amounts of monoclonal antibodies at 4°C for 30 min in the dark (Supplemental Material). The labeled cells were then washed twice with PBS containing 2% FBS, 1mM EDTA before data acquisition. Data acquisition was performed using an LSRFortessa (BD Biosciences, CA, USA) using a high throughput loader system. Data was acquired using FACSDiva software (BD Biosciences) and analyzed with FlowJo version X software (TreeStar Inc. OR, USA). NK cells were gated as live CD56^+^CD3^-^CD19^-^CD14^-^, T cells were gated as live CD3^+^CD56^-^CD19^-^CD14^-^ followed by CD4/CD8 and CD45RA/CD45RO comparison to identify naïve/effector T cells and memory T cells, respectively (S2 Fig). Data was plotted by GraphPad Prism version VI (GraphPad Software Inc. La Jolla, CA, USA) or FlowJo version X software (TreeStar Inc.). A complete overview about antibody clones and used panels, see S1 Table to S5 Table.

### Statistical analyses

The mean, median, and range of Dara infusions, inclusion age and percentage of infusion-related reactions and reduction of M-component were calculated using the descriptive statistics functions of Excel. Figures were plotted using Excel or GraphPad Prism, and most figures show individual patients. Time to event, progression free survival and overall survival were calculated using Kaplan-Meier analysis with Statistica.

## Results

### Patients and treatment

Of the 23 patients included, three patients received Dara as a part of early access program supplied by Genmab A/S, while the remaining 20 patients received Dara as a part of the single patient request program, provided by *Janssen-Cilag AB*. Patients received a median of nine Dara infusions, with a range of two to 34 infusions of Dara. All patients had exhausted all available treatment options and had advanced MM with resistance to previously used anti-myeloma drugs. The median inclusion age was 63 (34–82) years. Previous treatment and patient’s characteristics are summarized in Table 1.

**Fig 1:**
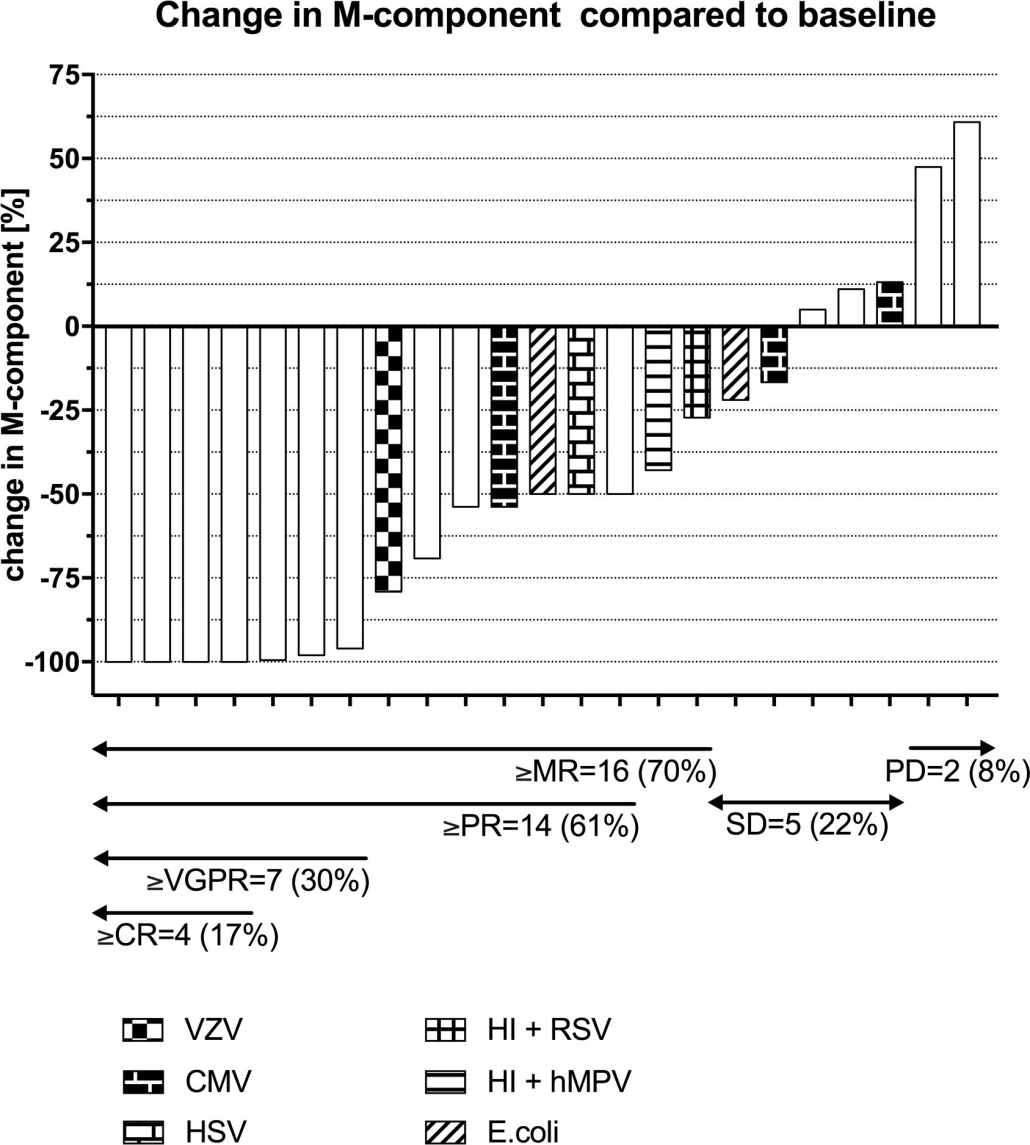
Waterfall plot of changes of M-component in response to dara treatment. The changes in measurable M-component are plotted and grouped according to IMWG treatment response criteria into CR = complete remission, VGPR = very good partial remission, PR = partial remission, MR = minimal response, SD = stable disease and PD = progressive disease. Bacterial and viral infections are shown for nine patients that suffered from infectious complications. VZV = varicella zoster virus, UTI = urinary tract infection, EBV = Epstein-Barr virus, HI = H. Influenza, hMPV = human metapneumovirus, RSV = respiratory syncytial virus, CMV = cytomegalovirus.

In line with previously published studies, Dara treatment showed high efficacy and safety [2,3,5]. In total, fourteen patients (61%) showed a response to treatment according to the response criteria of the International Myeloma Working Group (IMWG) guidelines. Four patients achieved complete remission (CR), three very good partial remissions (VGPR), and seven patients achieved partial remission (PR), two patients showed minimal response (MR), five patients had stable disease (SD) and only two patients had progressive disease (PD) on treatment (Fig 1). The median overall survival was 9.5 months, and the median progression-free survival was four months. Infusion-related reactions (IRRs) of grade 2 and below were observed in sixteen patients (70%). The symptoms included rhinal congestion, coughing, buccal irritation, dyspnea, fever and nausea. All IRRs occurred during the first infusion. IRRs were safely managed with pre- and post-infusion medications consisting of antihistamine, antipyretic analgesic and corticosteroid. Of four patients with chronic kidney disease (CKD) at the time of inclusion (two patients with stage 3, one patient with stage 4 and one patient with stage 5 CKD, see Table 1), one patient achieved PR, two patients MR (with 27% and 43% reduction of M-component) and one patient SD. The patient with stage 5 CKD was hospitalized during the first infusion for monitoring of metabolic balance. No noninfectious Dara related complications were noticed in patients with CKD.

**Fig 2:**
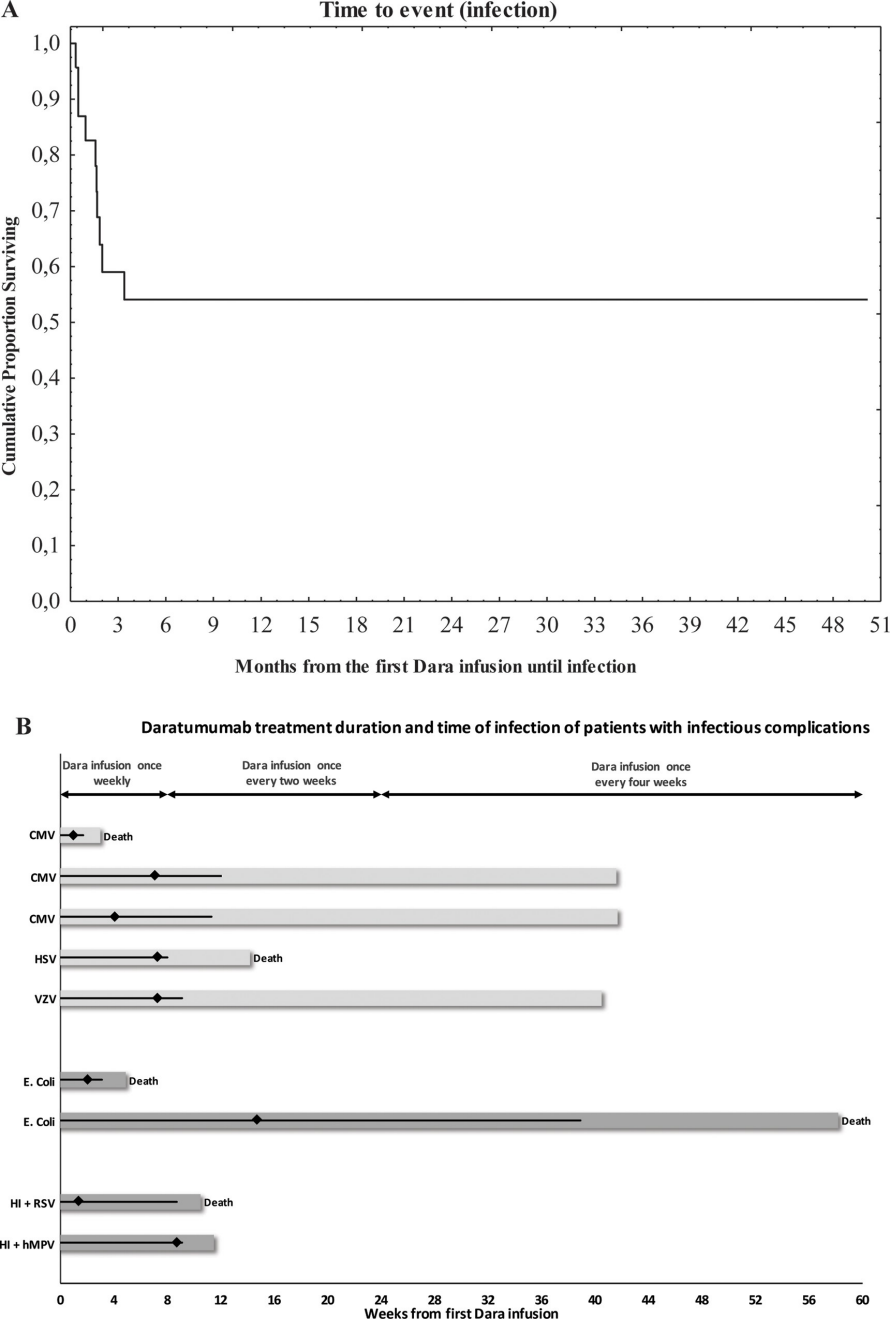
Timeline of dara treatment and occurrence of infectious complications of all patients. Dara treatment was given according to protocol, as indicated in the experimental procedures and study design. Nine of 23 patients suffered from bacterial or viral infections, the median time to event are presented in Fig 2A. The unique timeline of Dara treatment is depicted for each of the nine patients with infectious complications, as shown in Fig 2B, with the time of total Dara treatment as a black line, and the occurrence of infection with a black rhomb. Dark grey bars indicate bacterial infections, and light grey bars indicate viral complications. End of follow-up due to death is noted in the figure. The insert shows the median and range of weeks of Daratumumab treatment and follow-up. CMV = cytomegalovirus, HI = H. Influenza, RSV = respiratory syncytial virus, hMPV = human metapneumovirus, HSV = herpes simplex virus, VZV = varicella zoster virus.

The median follow-up time (from first Dara infusion) for all patients was eight months (CI one—eight months), and for patients with infections, it was three months (CI one—ten months). All events (infections) occurred at a median of 49 days (CI 15–61 days) (Fig 2A).

### Infections

A total of nine (39%) patients suffered from infections, two (9%) from bacterial infections only, five (22%) from viral infections and two (9%) from both viral and bacterial infections (Figs 1 and 2). Interestingly, all patients that suffered from bacterial or viral infections or reactivations were classed as PR, MR or SD, based on the changes in M-component according to IMWG criteria (Fig 1). Furthermore, none of the patients with a CR or VGPR suffered from infectious complications in our study (Fig 1). The majority of infections occurred in the first ten weeks of treatment (Fig 2B). The viral infections consisted of exogenous infections in two (9%) patients and reactivation of latent infections in five (22%) patients. All patients (except one, viral encephalitis) received antiviral prophylaxis with Aciclovir 250mg twice daily.

#### Bacterial infections

Out of the four patients with bacterial infections, two patients suffered from symptomatic urinary tract infections (*E*. *coli*) and two from pneumonia (*H*. *influenza*). One case of pneumonia was fatal, where the patient developed acute heart failure. This was judged as being not related to Dara treatment. The other three cases resolved with antibacterial treatment. The frequencies of NK cells (CD3^-^CD56^+^), immature NK cells (CD56^+^NKG2A^+^) and mature NK cells capable of performing ADCC (CD56^+^CD16^+^) of one patient that suffered from *E*. *coli* infection are presented in S1 Fig.

#### Exogenous viral infections

One case with respiratory syncytial virus (RSV) and one case with human metapneumovirus (hMPV) was observed in conjunction with infection with E. coli (Figs 1 and 2), both patients recovered without consequences.

#### Reactivated latent infections

Among the five patients with viral reactivations, three cases of cytomegalovirus where observed, one of these resolved with antiviral treatment, one case resolved without treatment and in one case the patient developed severe heart failure and subsequently died. One patient developed localized zoster (varicella zoster virus), which resolved with antiviral treatment, and we observed one case of encephalitis (herpes simplex virus), which was fatal.

#### Herpes simplex virus (HSV)

**Fig 3:**
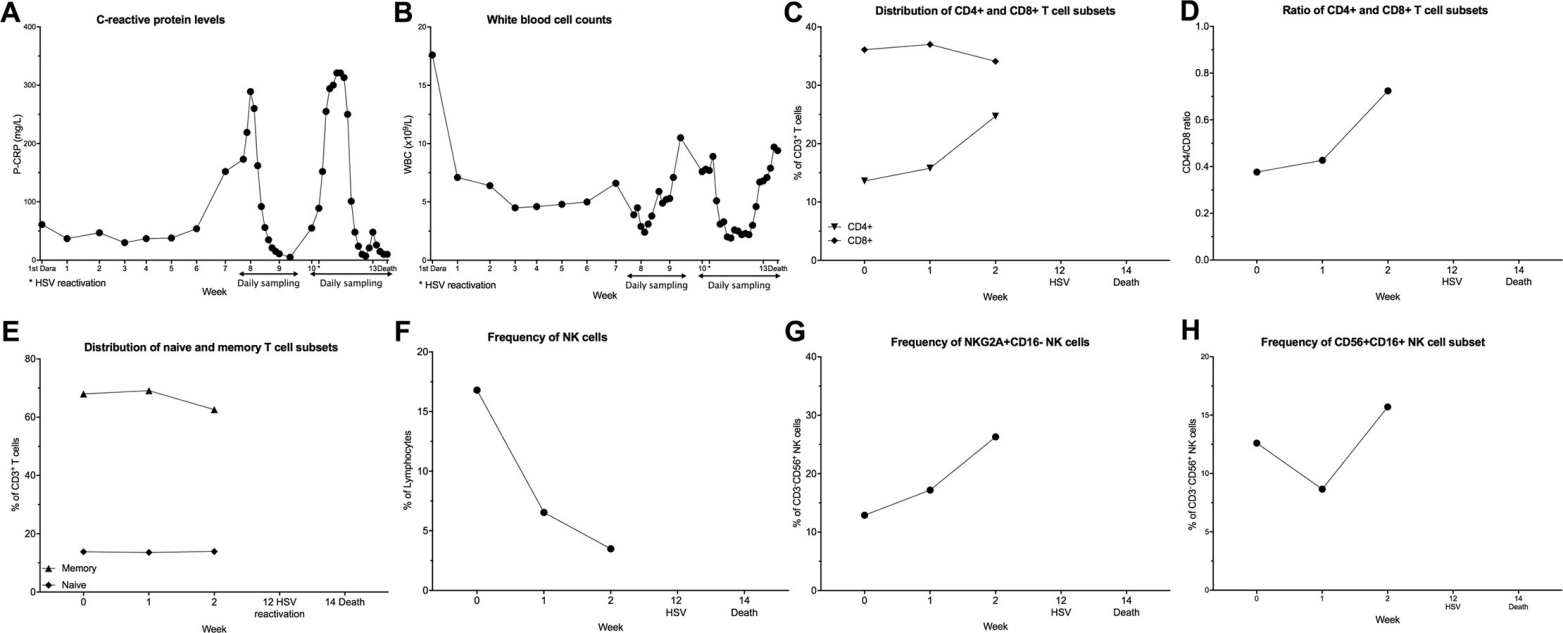
Clinical and immunological parameters from a patient with HSV infection during dara treatment. (A-B): The laboratory parameters CRP (A) and WBC at admission during the HSV-1 reactivation until the death of the patient are shown. (C-H) Peripheral blood samples were collected from patients throughout the Dara treatment. PBMCs were stained with lymphoid lineage markers and analyzed by flow cytometry, as described in experimental procedures. Frequencies of T cell and NK cells subsets are depicted. (C) percentages of CD8^+^ and CD4^+^ T cells and (D) CD4/CD8 ratio during Dara treatment (E) distribution of memory (CD45RA^-^CD45RO^+^) and naïve (CD45RA^+^CD45RO^-^) T cell subpopulation. In (F), total CD3^-^CD56^+^ NK cells as the percentage of lymphocytes and (G) CD56^+^CD16^-^NKG2A^+^ NK cells as the percentage of CD3^-^CD56^+^ NK cells are shown. (H) shows frequency of CD56^+^CD16^-^ as the percentage of total CD3^-^CD56^+^ NK cells.

One patient, who received a total of nine doses of Dara and responded with PR, presented with reactivated HSV infection. Before administration of the 10^th^ Dara dose, the patient was admitted due to confusion, and a subsequent cranial CT-scan demonstrated lesions attributable to HSV, which was confirmed with HSV PCR of cerebrospinal fluid. The patient responded to antiviral treatment (both the CRP and PCR normalized) started at the day of hospitalization, but never regained consciousness and died from multi-organ failure seventeen days post admission. The CRP levels were elevated up to 320 mg/L in the week before HSV detection (Fig 3A). After this initial increase, CRP decreased back to 10 mg/L but increased again. This increase in CRP parallels a decrease of white blood cell (WBC) count (Fig 3B). A phentotypic analysis of immune cells revealed a low frequency of CD4^+^ T cells, which increased slightly during the treatment, while there was no major change in the level of CD8^+^ T cells (Fig 3C). The low amount of CD4^+^ T cells resulted in a very low CD4/CD8 ratio, which can be used as a measurement of normal T cell distribution (Fig 3D). Ratios between 1.5–2.5 are generally considered normal, and a low or inverted CD4/CD8 ratio is associated with altered immune functions such as seen in infections and chronic inflammation [20].

Interestingly the percentage of memory T cells (CD45RA^-^CD45RO^+^) among total CD3^+^ T cells is very high (70%) and stable during treatment, while consequently, the naïve T cell subpopulation is stable at low percentages (13–14%) (Fig 3E). In the NK cell compartment, a drastic decrease in circulating NK cells (CD3^-^CD56^+^) shortly after the start of Dara treatment could be observed (Fig 3F), similar to previously published results [7]. Although there is a continuous decrease in total NK cells, the frequencies of immature CD56^+^NKG2A^+^CD16^-^ and CD56^+^CD16^+^ NK cell subpopulations increased in the first two weeks after the start of Dara treatment (Fig 3G and 3H).

#### Cytomegalovirus (CMV)

**Fig 4:**
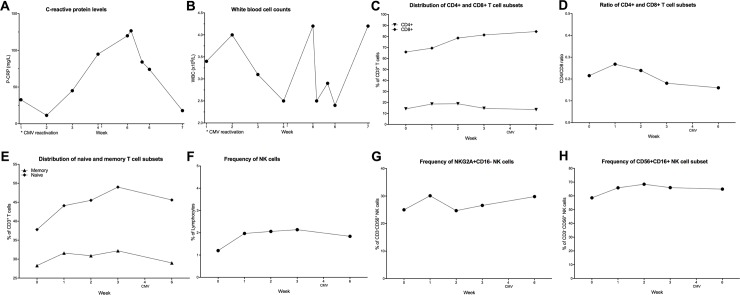
Clinical and immunological parameters from a 65y male patient with CMV reactivation during dara treatment. (A-B): The laboratory parameters CRP (A) and WBC at admission during the Dara treatment and CMV reactivation are shown. (C-H) Peripheral blood samples were collected from patients throughout the Dara treatment. PBMCs were stained with lymphoid lineage markers and analyzed by flow cytometry, as described in experimental procedures. Frequencies of T cell and NK cells subsets are depicted. (C) percentages of CD8^+^ and CD4^+^ T cells and (D) CD4/CD8 ratio during Dara treatment (E) distribution of memory (CD45RA^-^CD45RO^+^) and naïve (CD45RA^+^CD45RO^-^) T cell subpopulation. In (F), total CD3^-^CD56^+^ NK cells as the percentage of lymphocytes and (G) CD56^+^CD16^-^NKG2A^+^ NK cells as the percentage of CD3^-^CD56^+^ NK cells are shown. (H) shows frequency of CD56^+^CD16^-^ as the percentage of total CD3^-^CD56^+^ NK cells.

Of 23 patients that were monitored for CMV, three patients showed reactivation of CMV by PCR analysis. The first patient was a 65-y old male who developed fever and upper respiratory tract infection. PCR assessment showed a CMV burden of, 26000 IU/mL, and Dara was discontinued during antiviral treatment with valganciclovir. CMV copy numbers decreased to 500 IU/ml after ten days, and the patient was subsequently restarted on Dara. CRP level and WBC count increase before CMV reactivation (Fig 4A and 4B). An increase of CD8^+^ T cells at the beginning of Dara treatment could be observed while there was no change in CD4^+^ T cell frequency (Fig 4C). This high percentage of CD8^+^ T cells and the low frequency of CD4^+^ T cells resulted in a low CD4/CD8 ratio (Fig 4D). There was an increase in both the in naive T cells (CD45RA^+^CD45RO^-^) and memory T cells (CD45RA^-^CD45RO^+^) detectable during Dara treatment, but interestingly, the frequencies both populations dropped in the weeks before CMV reactivation (Fig 4E). Already at the beginning of the Dara treatment, the NK cell population was very low and remained at low levels during treatment (Fig 4F). The levels of NKG2A^+^CD16^-^ increased slightly before detection of CMV (Fig 4G), while the CD16^+^ NK cells did not change before or after CMV reactivation (Fig 4H).

**Fig 5:**
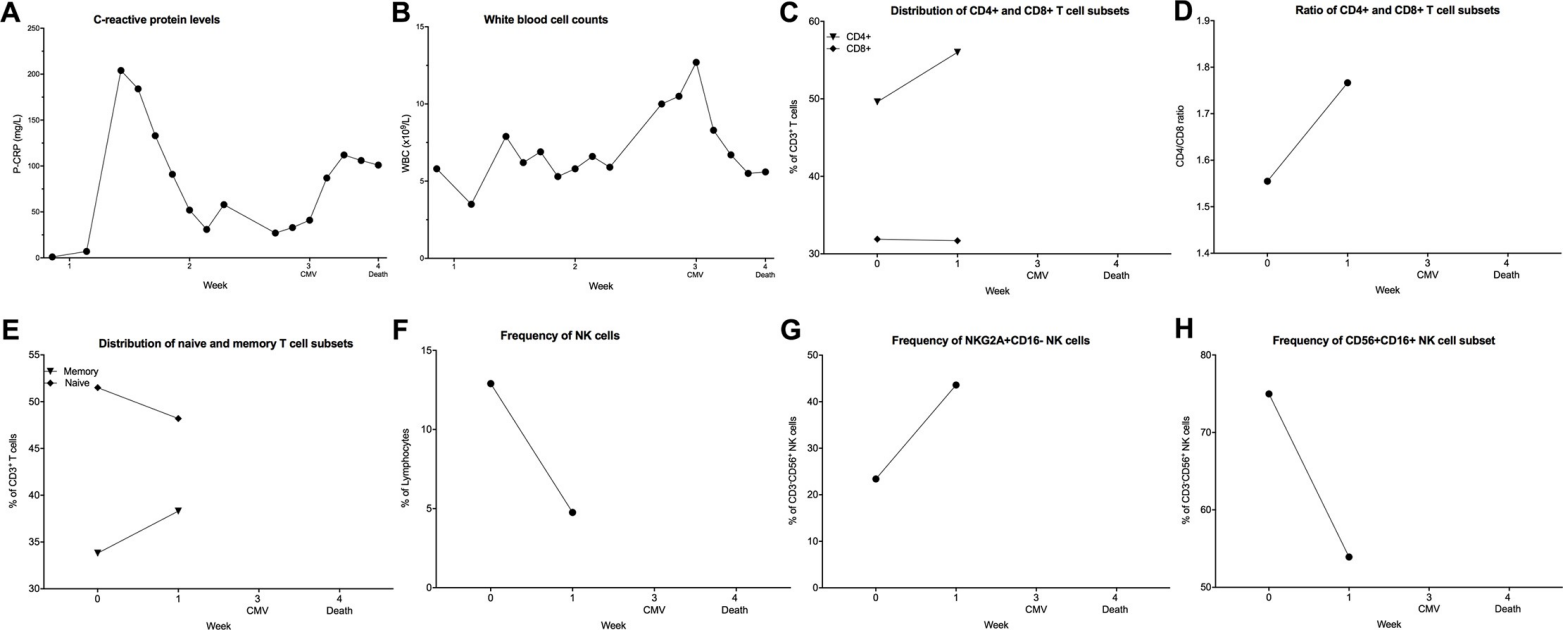
Clinical and immunological parameters from an 82y male patient with CMV reactivation during dara treatment. (A-B): The laboratory parameters CRP (A) and WBC at admission during the Dara treatment and CMV reactivation until the death of the patient are shown. (C-H) Peripheral blood samples were collected from patients throughout the Dara treatment. PBMCs were stained with lymphoid lineage markers and analyzed by flow cytometry, as described in experimental procedures. Frequencies of T cell and NK cells subsets are depicted. (C) percentages of CD8^+^ and CD4^+^ T cells and (D) CD4/CD8 ratio during Dara treatment (E) distribution of memory (CD45RA^-^CD45RO^+^) and naïve (CD45RA^+^CD45RO^-^) T cell subpopulation. In (F), total CD3^-^CD56^+^ NK cells as the percentage of lymphocytes and (G) CD56^+^CD16^-^NKG2A^+^ NK cells as the percentage of CD3^-^CD56^+^ NK cells are shown. (H) shows frequency of CD56^+^CD16^-^ as the percentage of total CD3^-^CD56^+^ NK cells.

A second patient with CMV reactivation, an 82y old male, developed hepatic insufficiency after receiving two doses of Dara and was admitted. CMV copy number at the time of admission was 2600 IU/mL. The patient died, and the post-mortem autopsy revealed severe heart failure as a possible cause of death. CRP and WBC levels show a dramatic increase before CMV reactivation (Fig 5A and 5B). The frequency of CD8^+^ T cells was stable while the CD4^+^ T cell population increased (Fig 5C), leading to an increase in the CD4/CD8 ratio (Fig 5D). The frequency of memory T cells (CD45RA^-^CD45RO^+^) increases during the first two weeks of Dara treatment (Fig 5E). Although the NK cell percentage was decreasing drastically after Dara treatment (Fig 5F), the percentage of NKG2A^+^CD16^-^ subpopulation was slightly increased (Fig 5G) while the CD56^+^CD16^+^ population was decreased (Fig 5H).

The third patient, 80y male, contracted an upper respiratory tract infection after the 6^th^ dose of Dara, simultaneously CMV copy number increased to 800 IU/mL. The respiratory tract infection resolved spontaneously two weeks after withdraws of Dara (without CMV treatment), and the patient could be restarted with Dara. We were not able to conduct an in-depth flow cytometric analysis of the immune cells for this patient.

#### Varicella zoster virus (VZV)

**Fig 6:**
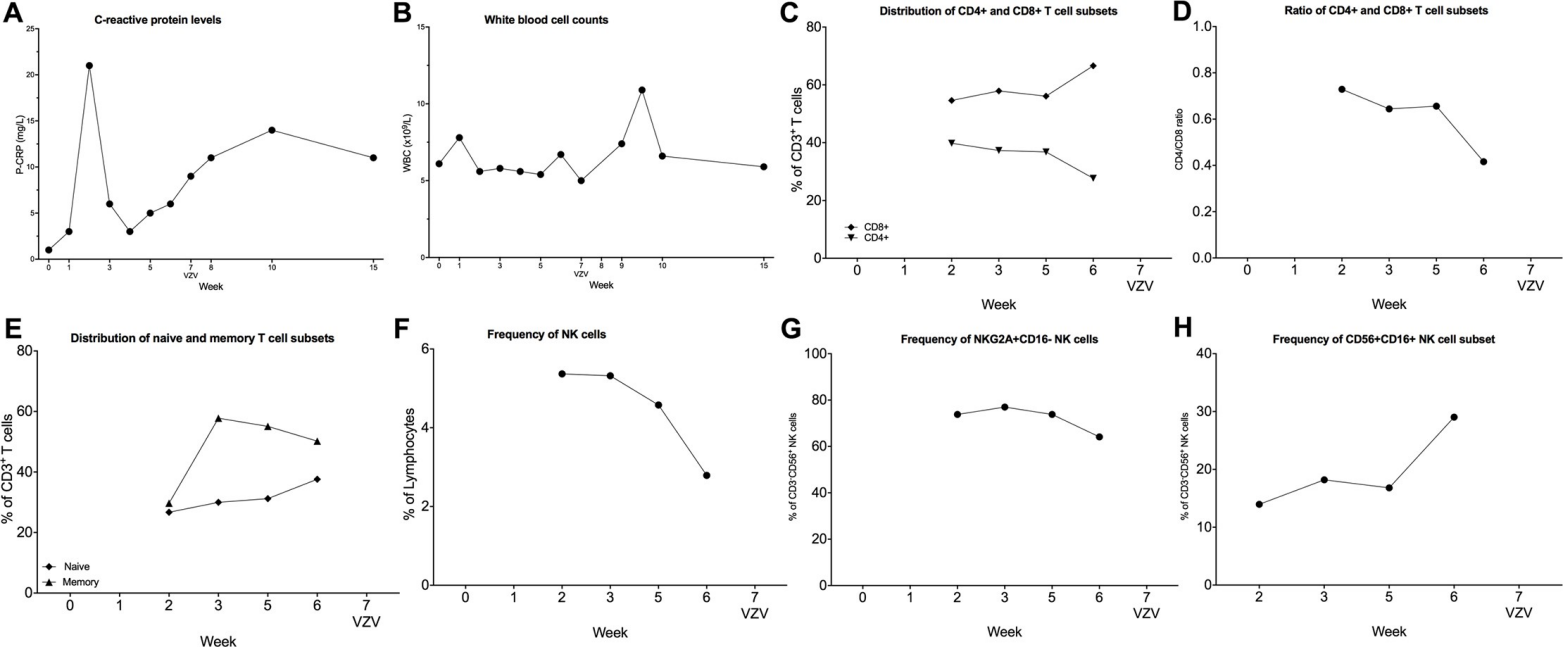
Clinical and immunological parameters from a patient with VZV reactivation during dara treatment. (A-B): The laboratory parameters CRP (A) and WBC at admission during the Dara treatment and VZV reactivation are shown. (C-H) Peripheral blood samples were collected from patients throughout the Dara treatment. PBMCs were stained with lymphoid lineage markers and analyzed by flow cytometry, as described in experimental procedures. Frequencies of T cell and NK cells subsets are depicted. (C) percentages of CD8^+^ and CD4^+^ T cells and (D) CD4/CD8 ratio during Dara treatment (E) distribution of memory (CD45RA^-^CD45RO^+^) and naïve (CD45RA^+^CD45RO^-^) T cell subpopulation. In (F), total CD3^-^CD56^+^ NK cells as the percentage of lymphocytes and (G) CD56^+^CD16^-^NKG2A^+^ NK cells as the percentage of CD3^-^CD56^+^ NK cells are shown. (H) shows frequency of CD56^+^CD16^-^ as the percentage of total CD3^-^CD56^+^ NK cells.

A 62y old female, with prior treatments including Allo-SCT, was started on Dara treatment. Despite ongoing antiviral prophylaxis (aciclovir 250mg twice daily), the patient experienced VZV reactivation that responded to aciclovir 500mg twice daily, after which Dara treatment could be continued. The CRP levels increased in the second week of Dara treatment, then returned to low values and then increased again around the time of VZV reactivation (Fig 6A). The WBC counts were stable with a small peak after VZV reactivation (Fig 6B). CD4^+^ T cells decrease slowly during Dara treatment while CD8^+^ T cell frequencies increase before VZV reactivation (Fig 6C), which results in low CD4/CD8 ratio (Fig 6D). Although the NK cell percentage was still within a normal range for the first two weeks after the initial Dara infusion, they decreased during the treatment (Fig 6F). The percentage of NKG2A^+^CD16^-^ subpopulation was stable (Fig 6G) while the CD56^+^CD16^+^ population increased five weeks after the start of Dara treatment and peaked in the week before VZV reactivation (Fig 6H).

#### Allogeneic transplantation

Two patients, who had received Allo-SCT, were included in the study. The first patient was the 62y old female who relapsed after Allo-SCT and suffered from VZV reactivation during Dara treatment (Fig 6). Although she achieved a PR initially during Dara treatment, this response was short-lived (six months), and she progressed while she was still on treatment. The second patient, a 34y old male, who was treated with Allo-SCT upfront, followed by an aggressive relapse including extramedullary plasmacytoma, received Dara and subsequently achieved a CR until a second Allo-SCT from the same sibling donor. The patient was still on monthly maintenance treatment ten months after the second transplant. None of the patients developed graft versus host disease (GVHD) on treatment with Dara. The 34y old male suffered no infections or reactivation of latent infections.

**Fig 7:**
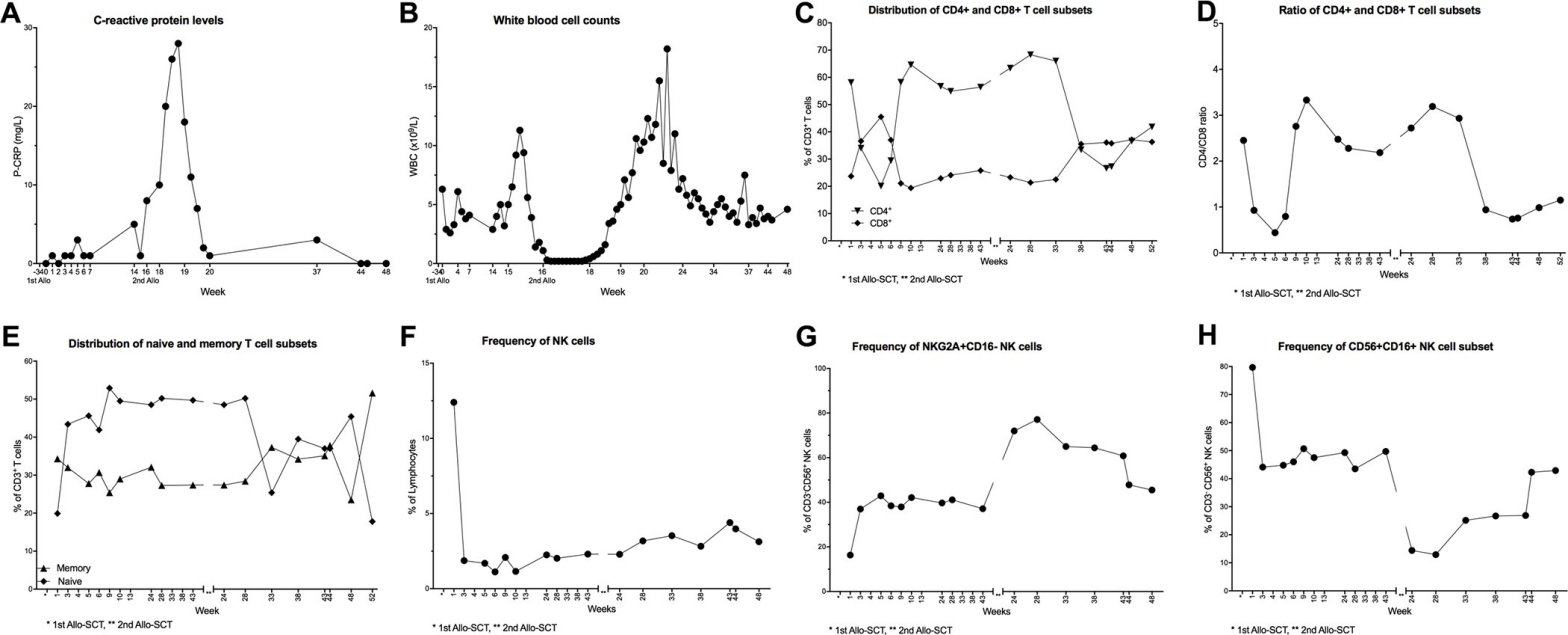
Clinical and immunological parameters from a patient who received Allo-SCT and dara treatment. (A-B): The laboratory parameters CRP (A) and WBC at admission during the Dara treatment are shown. (C-H) Peripheral blood samples were collected from patients throughout the Dara treatment. PBMCs were stained with lymphoid lineage markers and analyzed by flow cytometry, as described in experimental procedures. Frequencies of T cell and NK cells subsets are depicted. (C) percentages of CD8^+^ and CD4^+^ T cells and (D) CD4/CD8 ratio during Dara treatment (E) distribution of memory (CD45RA^-^CD45RO^+^) and naïve (CD45RA^+^CD45RO^-^) T cell subpopulation. In (F), total CD3^-^CD56^+^ NK cells as the percentage of lymphocytes and (G) CD56^+^CD16^-^NKG2A^+^ NK cells as the percentage of CD3^-^CD56^+^ NK cells are shown. (H) shows frequency of CD56^+^CD16^-^ as the percentage of total CD3^-^CD56^+^ NK cells.

The CRP and WBC levels increased dramatically after the second Allo-SCT (Fig 7A and 7B). The frequencies of CD4^+^ and CD8^+^ T cells fluctuated (Fig 7C). When we looked at the CD4/CD8 ratio in the patient that received Allo-SCT, we could observe a dramatic change in the frequencies of CD4^+^ and CD8^+^ T cells after the first Dara treatment, leading to an inverted CD4/CD8 ratio of 0.44 (Fig 7D), which is below the normal range [20]. After six weeks, the CD4/CD8 ratio had stabilized again at a value of 3.36. The patient was on Dara treatment while he received a second Allo-SCT from the same sibling donor, and again, an inverted CD4/CD8 ratio could be observed.

Similarly, fluctuations in the pool of memory (CD45RA^-^CD45RO^+^) and naïve (CD45RA^+^CD45RO^-^) T cell pools could be observed (Fig 7E). In line with our previous findings in patients receiving autologous SCT [7], NK cell frequencies are going down drastically from 12.4% to under 2% during the first week after the first dose of Dara (Fig 7F). Initially the frequency of CD56^+^NKG2A^+^ NK cells, on the other hand, is doubling (20% -> 40%) after 1^st^ Dara cycle and is increasing even further (80%) at the beginning of the 2^nd^ treatment cycle followed by a slow decrease to the levels prior the 2^nd^ Dara cycle (Fig 7G). The percentage of CD56^+^CD16^+^ NK cells is decreasing at the same rate as the CD56^+^NKG2A^+^CD16^-^ NK cells are increasing (Fig 7H). This could be indicative of a shift from an NK cell population that has strong ADCC activity towards a more immature NK cell pool that is CD16^-^ and thus not able to bind to Dara and perform ADCC.

## Discussion

The efficacy of Dara monotherapy in patients with relapsed or refractory MM has made it a promising new agent for immunotherapy. Dara is a first-in-class anti-CD38 humanized antibody, that exerts its activity via a variety of mechanisms, including ADCC [21] NK cells, the major mediators of ADCC, also express CD38. The CD38 levels on NK cells are even increased when the cells become activated. Consequently, Dara treatment results in a depletion of NK cells and other CD38-expressing immune cells during Dara treatment [6,7]. NK cells are innate immune cells that quickly respond to infections by intracellular pathogens, and genetic deficiencies and the depletion of this cell population is associated with recurrent infections [22].

In this study, a cohort of 23 patients received Dara as a single-agent therapy, four of whom presented with CKD, and two patients received Dara in relapse after Allo-SCT. The patients were closely monitored for the occurrence of infections and nine out of the 23 patients suffered from infectious complications. Five of these cases were viral reactivations of the herpesvirus family. Dara showed durable single-agent activity with responses in 61% (≥PR) of the patients which is higher compared to previously published results [8,23,24].In the previous studies, the patients had undergone a median of 5 prior lines of treatment compared to three prior lines in our study. None of the patients had alternative effective treatment options. The novel target and unique mechanisms of action of Dara differentiate it from existing therapies. Given the high and unmet medical need in relapsed and refractory MM and the lack of alternative therapies, Dara provides an attractive option with a potentially high benefit/risk profile in this patient population.

Most of the relapse/refractory patients undergoing MM therapy are on antiviral prophylaxis as per international and national recommendations. This is expected to be the case in most of the patients in the recently published phase III trials with Dara (recommended by protocol), making it difficult to speculate on the prevalence of HSV and VZV reactivation in patients not receiving prophylaxis [3,5]. It is evident that immune responses against HSV-1 and HSV-2 are complex and involve a delicate interplay between innate and adaptive immune compartments. Studies on the role of NK cells in HSV infections have yielded contradicting results in experimental systems [25–27]. Among others, these reports indicate the complexity of the antiviral cellular responses [28]. HSV encephalitis is a rare condition, and there is evidence that HSV encephalitis (at least in children) has a genetic predisposition as a monogenetic inborn error of immunity [29]. In this study, we identified one patient, without antiviral prophylaxis, with HSV encephalitis that died despite antiviral treatment.

While screening for CMV infections, we identified three (13%) patients with CMV reactivations, of which two were treated, and one patient whose reactivation resolved without antiviral therapy after a short period of Dara withdrawal. Thus, reactivation of latent CMV is not infrequent during treatment with Dara even with prophylactic antiviral treatment. Surprisingly, a recently published study, where data on 148 patients from the GEN501 and SIRIUS clinical trials were combined [2,3], showed a very low occurrence of infectious complications [6] The authors report that patients suffering from infectious complications (55% of patients) primarily suffered from upper respiratory tract infection, nasopharyngitis, pneumonia and sinusitis. Only 2% of patients got herpes zoster, and no cases of CMV or HSV were reported [6].

It can only be speculated as to why the results in respect to reactivations of the Herpesviridae family in this study differs from ours. In our unit, all patients are screened very carefully for CMV during treatment, although the three cases of CMV were symptomatic, this could be an explanation why we report a higher incidence of CMV compared to previous studies [2,3,6]. While GEN501 included patients from Scandinavia where seroprevalence of CMV is relatively high, 83% [30] in the adult Swedish population, SIRIUS included mostly patients from USA and Canada where seroprevalence is more variable (67–87% in the USA) [31,32].

Overall, both previous studies and our study show a high incidence of infectious complications.

It should be recognized that CMV reactivations frequently recover without antiviral therapy in most patients with hematological diseases except for allogeneic stem cell transplant recipients. In a phase III trial in chronic lymphocytic leukemia (CLL) comparing alemtuzumab (anti-CD52) to chlorambucil, the risk of developing PCR-positive CMV in the alemtuzumab arm was 69%, with 15.6% of these patients displaying symptomatic CMV infections without end organ involvement. Among the 78 asymptomatic alemtuzumab patients who had one or more PCR-positive CMV results, treatment was interrupted in 47, and only 36 received antiviral treatment [33]. Current international recommendations do not recommend routine monitoring of multiple myeloma patients [34]. The findings in our study support a need for more thorough screening of patients for viral reactivations.

Interestingly, in three patients that suffered from herpesvirus reactivation (CMV, HSV, VZV), a very low ratio of CD4^+^ helper T cells to CD8^+^ cytotoxic T cells (CD4/CD8 ratio) was detected. The CD4/CD8 ratio, although showing a wide heterogeneity in healthy individuals, can be used to predict altered immune functions [20]. Ratios between 1.5–2.5 are generally considered normal, and a low or inverted CD4/CD8 ratio is associated with altered immune functions such as seen in infections and chronic inflammation [20]. It has been previously reported that CD38^+^ Tregs are depleted during Dara treatment, resulting in a decreased CD4/CD8 ratio [35]. However, we were not able to assess whether the low CD4/CD8 ratios seen in our patients was a result of a preferential expansion of the CD8^+^ T cell population or reduced survival of the CD4^+^ T cells.

Five out of 23 patients treated with Dara suffered from reactivation with viruses of the Herpesviridae family. Given that these patients had advanced disease and were heavily pre-treated, infectious complications could reflect a combination of decreased innate responses and dysfunctional humoral and cell-mediated immunity, already present in these patients. It is well known that NK cells contribute to the defense against herpesviruses, including CMV, HSV-1 and VZV [27,36–39]. NK cells can detect virus infection directly by recognition of viral proteins, and indirectly by binding to molecules that are up-regulated on stressed cells. Furthermore, once a primary infection has led to the activation of the adaptive immune system and the generation of virus-specific antibodies, NK cells can also kill infected cells via ADCC [27].

Recent studies implicate that balance of activation and downregulation of NK-cells as a control form for viral infections. Correlation of seropositivity for CMV with increased expression of activating receptor NKG2C has previously been described [40], with NKG2C^+^ impacting cytotoxicity on HLA-E expressing cells such as haematological malignancies which have been shown to affect myeloma cells [41]. In this study, we found increased levels of the inhibitory receptor NKG2A in one of two CMV seropositive patients but also in the patient affected by HSV and the patient undergoing double Allo-SCT. Further studies are needed to establish whether this difference from previous studies in CMV seropositive patients is due to Dara treatment.

In several clinical trials, it has been shown that Dara treatment shows very good efficacy both as single agent treatment or in combination treatments [3,42]. However, we and others have previously published that NK cells specifically disappear upon treatment of MM with Dara [7] and this will impede the immune system’s ability to fight the disease. Of interest, a recent publication by *Wang et al*. examined the underlying mechanisms of Dara on NK cells. It was shown that NK cells that remain after Dara administration show a specific phenotype of CD38^-/low^. Due to the expression of CD38 on NK cells, it is possible that NK cells commit fratricide via ADCC and thus are effector cell and target cell at the same time [23]. This would explain the loss of NK cell with high CD38 expression and survival of the CD38^-/low^ population,

In this study, our findings are consistent with previously published data on efficacy of Dara in the refractory MM patients [2]. It is known that Dara induces effective lysis of MM cells not only via ADCC but also via CDC, via apoptosis upon cross-linking with anti-immunoglobulin G antibody and by blocking the enzymatic activity. This study indicates the need for further prospective studies to assess the correlation between viral reactivation and peripheral blood cytotoxic lymphocyte counts. Due to Dara’s mechanism of action which heavily depends on ADCC NK cells in these patients could synergize the anti-myeloma effects of Dara through their activation. Therefore, the therapeutic perspectives of multidrug-refractory patients may be improved by combination therapies based on Dara.

## Supporting information

S1 Fig Immunological parameters from a patient with E.coli infection during dara treatment. (TIFF)

S2 Fig Gating strategy for NK cell and T cell populations. (DOCX)

S1 Table Antibody panel for T cell subsets. (DOCX)

S2 Table Antibody panel 1 for NK cell subsets. (DOCX)

S3 Table Antibody panel 2 for NK cell subsets. (DOCX)

S4 Table Antibody panel 3 for NK cell subsets. (DOCX)

S5 Table Antibody panel 4 for NK cell subsets. (DOCX)

## References

[1] Yeap WH, Wong KL, Shimasaki N, Teo EC, Quek JK, et al (2016) CD16 is indispensable for antibody-dependent cellular cytotoxicity by human monocytes. Sci Rep 6: 34310 10.1038/srep34310 27670158 PMC5037471

[2] Lokhorst HM, Plesner T, Laubach JP, Nahi H, Gimsing P, et al (2015) Targeting CD38 with Daratumumab Monotherapy in Multiple Myeloma. N Engl J Med 373: 1207–1219. 10.1056/NEJMoa1506348 26308596

[3] Dimopoulos MA, Oriol A, Nahi H, San-Miguel J, Bahlis NJ, et al (2016) Daratumumab, Lenalidomide, and Dexamethasone for Multiple Myeloma. N Engl J Med 375: 1319–1331. 10.1056/NEJMoa1607751 27705267

[4] Mateos MV, Dimopoulos MA, Cavo M, Suzuki K, Jakubowiak A, et al (2018) Daratumumab plus Bortezomib, Melphalan, and Prednisone for Untreated Myeloma. N Engl J Med 378: 518–528. 10.1056/NEJMoa1714678 29231133

[5] Palumbo A, Chanan-Khan A, Weisel K, Nooka AK, Masszi T, et al (2016) Daratumumab, Bortezomib, and Dexamethasone for Multiple Myeloma. N Engl J Med 375: 754–766. 10.1056/NEJMoa1606038 27557302

[6] Casneuf T, Xu XS, Adams HC 3rd, Axel AE, Chiu C, et al (2017) Effects of daratumumab on natural killer cells and impact on clinical outcomes in relapsed or refractory multiple myeloma. Blood Adv 1: 2105–2114. 10.1182/bloodadvances.2017006866 29296857 PMC5728278

[7] Alici E, Chrobok M, Lund J, Ahmadi T, Khan I, et al (2016) Re-challenging with anti-CD38 monotherapy in triple-refractory multiple myeloma patients is a feasible and safe approach. Br J Haematol 174: 473–477. 10.1111/bjh.13776 26455823

[8] Lonial S, Weiss BM, Usmani SZ, Singhal S, Chari A, et al (2016) Daratumumab monotherapy in patients with treatment-refractory multiple myeloma (SIRIUS): an open-label, randomised, phase 2 trial. Lancet 387: 1551–1560. 10.1016/S0140-6736(15)01120-4 26778538

[9] Usmani SZ, Weiss BM, Plesner T, Bahlis NJ, Belch A, et al (2016) Clinical efficacy of daratumumab monotherapy in patients with heavily pretreated relapsed or refractory multiple myeloma. Blood 128: 37–44. 10.1182/blood-2016-03-705210 27216216 PMC4937359

[10] Blimark C, Holmberg E, Mellqvist UH, Landgren O, Bjorkholm M, et al (2015) Multiple myeloma and infections: a population-based study on 9253 multiple myeloma patients. Haematologica 100: 107–113. 10.3324/haematol.2014.107714 25344526 PMC4281323

[11] Mariani S, Coscia M, Even J, Peola S, Foglietta M, et al (2001) Severe and long-lasting disruption of T-cell receptor diversity in human myeloma after high-dose chemotherapy and autologous peripheral blood progenitor cell infusion. British Journal of Haematology 113: 1051–1059. 11442502 10.1046/j.1365-2141.2001.02871.x

[12] Teh BW, Harrison SJ, Worth LJ, Thursky KA, Slavin MA (2016) Infection risk with immunomodulatory and proteasome inhibitor-based therapies across treatment phases for multiple myeloma: A systematic review and meta-analysis. Eur J Cancer 67: 21–37. 10.1016/j.ejca.2016.07.025 27592069

[13] Nencioni A, Schwarzenberg K, Brauer KM, Schmidt SM, Ballestrero A, et al (2006) Proteasome inhibitor bortezomib modulates TLR4-induced dendritic cell activation. Blood 108: 551–558. 10.1182/blood-2005-08-3494 16537813

[14] Marchesi F, Mengarelli A, Giannotti F, Tendas A, Anaclerico B, et al (2014) High incidence of post-transplant cytomegalovirus reactivations in myeloma patients undergoing autologous stem cell transplantation after treatment with bortezomib-based regimens: a survey from the Rome transplant network. Transpl Infect Dis 16: 158–164. 10.1111/tid.12162 24215479

[15] Hasegawa T, Aisa Y, Shimazaki K, Ito C, Nakazato T (2016) Cytomegalovirus reactivation in patients with multiple myeloma. Eur J Haematol 96: 78–82. 10.1111/ejh.12551 25810117

[16] Lonial S, Dimopoulos M, Palumbo A, White D, Grosicki S, et al (2015) Elotuzumab Therapy for Relapsed or Refractory Multiple Myeloma. N Engl J Med 373: 621–631. 10.1056/NEJMoa1505654 26035255

[17] Rajkumar SV, Harousseau JL, Durie B, Anderson KC, Dimopoulos M, et al (2011) Consensus recommendations for the uniform reporting of clinical trials: report of the International Myeloma Workshop Consensus Panel 1. Blood 117: 4691–4695. 10.1182/blood-2010-10-299487 21292775 PMC3710442

[18] Levey AS, Bosch JP, Lewis JB, Greene T, Rogers N, et al (1999) A more accurate method to estimate glomerular filtration rate from serum creatinine: a new prediction equation. Modification of Diet in Renal Disease Study Group. Ann Intern Med 130: 461–470. 10075613 10.7326/0003-4819-130-6-199903160-00002

[19] Foundation NK (2002 (suppl 1)) K/DOQI Clinical Practice Guidelines for Chronic Kidney Disease: Evaluation, Classification and Stratification. Am J Kidney Dis 39: S1–S266. 11904577

[20] Bruno G, Saracino A, Monno L, Angarano G (2017) The Revival of an "Old" Marker: CD4/CD8 Ratio. AIDS Rev 19: 81–88. 28182620

[21] de Weers M, Tai YT, van der Veer MS, Bakker JM, Vink T, et al (2011) Daratumumab, a novel therapeutic human CD38 monoclonal antibody, induces killing of multiple myeloma and other hematological tumors. J Immunol 186: 1840–1848. 10.4049/jimmunol.1003032 21187443

[22] Orange JS (2002) Human natural killer cell deficiencies and susceptibility to infection. Microbes Infect 4: 1545–1558. 12505527 10.1016/s1286-4579(02)00038-2

[23] Wang YF, Zhang YB, Hughes T, Zhang JY, Caligiuri MA, et al (2018) Fratricide of NK Cells in Daratumumab Therapy for Multiple Myeloma Overcome by Ex Vivo-Expanded Autologous NK Cells. Clinical Cancer Research 24: 4006–4017. 10.1158/1078-0432.CCR-17-3117 29666301 PMC6095810

[24] Yeap WH, Wong KL, Shimasaki N, Teo ECY, Quek JKS, et al (2016) CD16 is indispensable for antibody-dependent cellular cytotoxicity by human monocytes. Scientific Reports 6. 10.1038/srep34310 PMC5037471 27670158

[25] Nandakumar S, Woolard SN, Yuan D, Rouse BT, Kumaraguru U (2008) Natural killer cells as novel helpers in anti-herpes simplex virus immune response. J Virol 82: 10820–10831. 10.1128/JVI.00365-08 18715907 PMC2573218

[26] Chmielarczyk W, Engler H, Ernst R, Opit U, Kirchner H (1985) Injection of Anti-thy-1.2 Serum Breaks Genetic Resistance of Mice against Herpes Simplex Virus. Journal of General Virology 66: 1087–1094. 10.1099/0022-1317-66-5-1087 2582081

[27] Dai HS, Caligiuri MA (2018) Molecular Basis for the Recognition of Herpes Simplex Virus Type 1 Infection by Human Natural Killer Cells. Front Immunol 9: 183 10.3389/fimmu.2018.00183 29483911 PMC5816072

[28] Chew T, Taylor KE, Mossman KL (2009) Innate and adaptive immune responses to herpes simplex virus. Viruses 1: 979–1002. 10.3390/v1030979 21994578 PMC3185534

[29] Casanova JL (2015) Severe infectious diseases of childhood as monogenic inborn errors of immunity. Proc Natl Acad Sci U S A 112: E7128–7137. 10.1073/pnas.1521651112 26621750 PMC4697435

[30] Olsson J, Kok E, Adolfsson R, Lovheim H, Elgh F (2017) Herpes virus seroepidemiology in the adult Swedish population. Immun Ageing 14: 10 10.1186/s12979-017-0093-4 28491117 PMC5424393

[31] Simanek AM, Dowd JB, Pawelec G, Melzer D, Dutta A, et al (2011) Seropositivity to cytomegalovirus, inflammation, all-cause and cardiovascular disease-related mortality in the United States. PLoS One 6: e16103 10.1371/journal.pone.0016103 21379581 PMC3040745

[32] Schmaltz HN, Fried LP, Xue QL, Walston J, Leng SX, et al (2005) Chronic cytomegalovirus infection and inflammation are associated with prevalent frailty in community-dwelling older women. J Am Geriatr Soc 53: 747–754. 10.1111/j.1532-5415.2005.53250.x 15877548

[33] Hillmen P, Skotnicki AB, Robak T, Jaksic B, Dmoszynska A, et al (2007) Alemtuzumab compared with chlorambucil as first-line therapy for chronic lymphocytic leukemia. J Clin Oncol 25: 5616–5623. 10.1200/JCO.2007.12.9098 17984186

[34] Ljungman P (2008) CMV infections after hematopoietic stem cell transplantation. Bone Marrow Transplant 42 Suppl 1: S70–S72. 18724309 10.1038/bmt.2008.120

[35] Krejcik J, Casneuf T, Nijhof IS, Verbist B, Bald J, et al (2016) Daratumumab depletes CD38+ immune regulatory cells, promotes T-cell expansion, and skews T-cell repertoire in multiple myeloma. Blood 128: 384–394. 10.1182/blood-2015-12-687749 27222480 PMC4957162

[36] Kim CK, Choi YM, Bae E, Jue MS, So HS, et al (2018) Reduced NK cell IFN-gamma secretion and psychological stress are independently associated with herpes zoster. PLoS One 13: e0193299 10.1371/journal.pone.0193299 29466462 PMC5821387

[37] Bjorkstrom NK, Svensson A, Malmberg KJ, Eriksson K, Ljunggren HG (2011) Characterization of natural killer cell phenotype and function during recurrent human HSV-2 infection. PLoS One 6: e27664 10.1371/journal.pone.0027664 22110712 PMC3216993

[38] Hammer Q, Romagnani C (2017) About Training and Memory: NK-Cell Adaptation to Viral Infections. Adv Immunol 133: 171–207. 10.1016/bs.ai.2016.10.001 28215279

[39] Etzioni A, Eidenschenk C, Katz R, Beck R, Casanova JL, et al (2005) Fatal varicella associated with selective natural killer cell deficiency. J Pediatr 146: 423–425. 10.1016/j.jpeds.2004.11.022 15756234

[40] Guma M, Angulo A, Vilches C, Gomez-Lozano N, Malats N, et al (2004) Imprint of human cytomegalovirus infection on the NK cell receptor repertoire. Blood 104: 3664–3671. 10.1182/blood-2004-05-2058 15304389

[41] Bigley AB, Rezvani K, Shah N, Sekine T, Balneger N, et al (2016) Latent cytomegalovirus infection enhances anti-tumour cytotoxicity through accumulation of NKG2C+ NK cells in healthy humans. Clin Exp Immunol 185: 239–251. 10.1111/cei.12785 26940026 PMC4955006

[42] Plesner T, Arkenau HT, Gimsing P, Krejcik J, Lemech C, et al (2016) Phase 1/2 study of daratumumab, lenalidomide, and dexamethasone for relapsed multiple myeloma. Blood 128: 1821–1828. 10.1182/blood-2016-07-726729 27531679 PMC5054695

