# Supplementary figures and images for: Infectious complications and NK cell depletion following daratumumab treatment of Multiple Myeloma
Source: PLoS One. 2019 Feb 13;14(2):e0211927. doi: 10.1371/journal.pone.0211927 (PMC6374018; doi:10.1371/journal.pone.0211927)

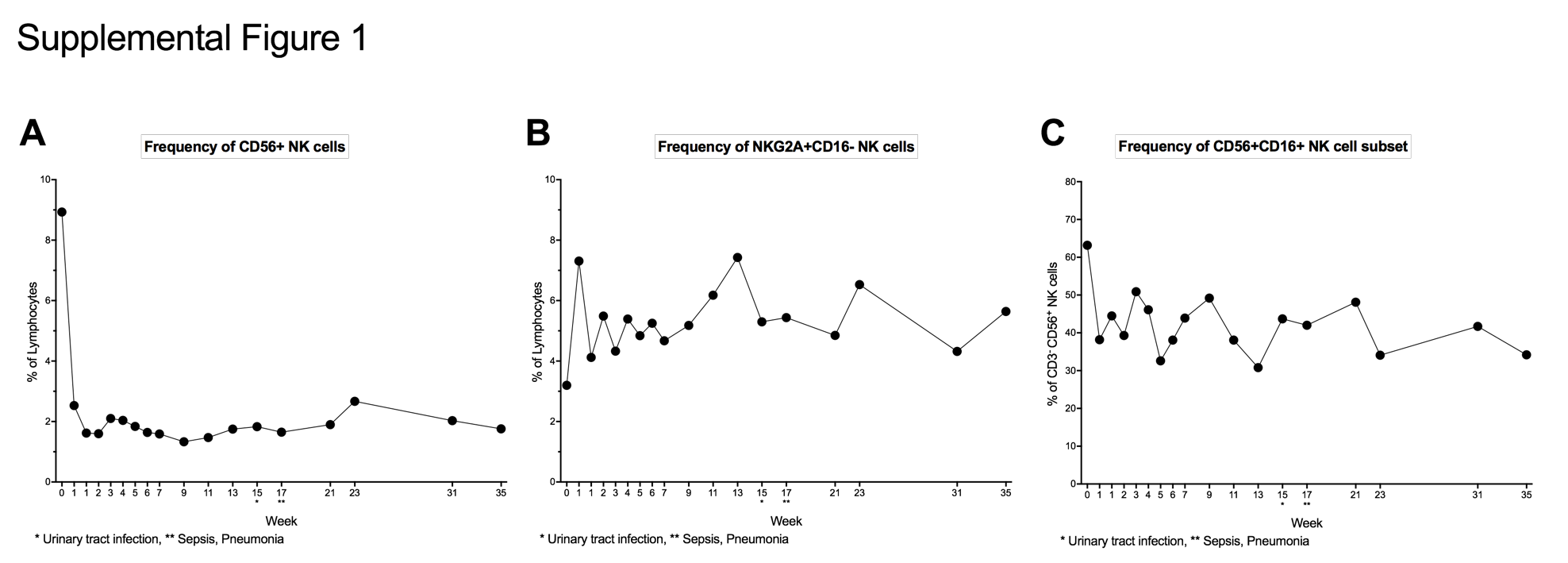

Supplement: S1 Fig — (TIFF) [file pone.0211927.s001.tiff]
