# Supplementary material for: Infectious complications and NK cell depletion following daratumumab treatment of Multiple Myeloma
Source: PLoS One. 2019 Feb 13;14(2):e0211927. doi: 10.1371/journal.pone.0211927 (PMC6374018; doi:10.1371/journal.pone.0211927)
Supplement: S2 Fig — (DOCX) [file pone.0211927.s002.docx]

**Supporting Information**

**S2 Fig: Gating strategy for NK cell and T cell populations.**

T cells were gated as live CD3^+^CD56^-^CD19^-^CD14^-^ followed by CD4/CD8 and CD45RA/CD45RO comparison to identify naïve/effector T cells and memory T cells, respectively. NK cells were gated as live CD56^+^CD3^-^CD19^-^CD14^-^.
