# Supplementary material for: Infectious complications and NK cell depletion following daratumumab treatment of Multiple Myeloma
Source: PLoS One. 2019 Feb 13;14(2):e0211927. doi: 10.1371/journal.pone.0211927 (PMC6374018; doi:10.1371/journal.pone.0211927)
Supplement: S1 Table — (DOCX) [file pone.0211927.s003.docx]

**Supporting Information**

**S1 Table: Antibody panel for T cell subsets.**

| **Laser** | **mAb Name** | **Conjugation** | **Clone** | **Company** |
| --- | --- | --- | --- | --- |
| Blue (488) | CD28 | FITC | CD28.2 | BD |
|  | 2B4 CD244 | PerCP-Cy5.5 | C1.7 | Biolegend |
| Yellow/Green (561) | CD152 | PE | BNI3 | BD |
|  | CD57 | PC-CF594 | NK-1 | BD |
|  | CD4 | Pe-Cy5 | RPA-T4 | BD |
|  | CD160 | PE-Cy7 | BY55 | Biolegend |
| Red (640) | CD8 | APC | RPA-T8 | BD |
|  | CD3 | Alexa Flour 700 | UCHT1 | BD |
|  | CD25 | APC-CY7 | M-A251 | BD |
| Violet (405) | PD1 CD279 | BV421 | EH12 | BD |
|  | CD14 | V500 | MP9 | BD |
|  | CD19 | V500 | HIB19 | BD |
|  | CD56 | BV510 | NCAM16.2 | BD |
|  | CD62L | BV605 | DREG-56 | BD |
|  | CD45RO | BV650 | UCHL1 | BD |
|  | CD45RA | BV785 | Hi100 | BD |

Vitally frozen PBMCs from all patients were thawed, washed and resuspended in cold PBS supplemented with 2% FBS and 1mM EDTA. Antibody stainings were performed by incubating the cells with monoclonal antibodies at 4°C for 30 min in the dark. The labeled cells were then washed twice with PBS containing 2% FBS, 1mM EDTA prior to data acquisition.
