# Supplementary material for: Infectious complications and NK cell depletion following daratumumab treatment of Multiple Myeloma
Source: PLoS One. 2019 Feb 13;14(2):e0211927. doi: 10.1371/journal.pone.0211927 (PMC6374018; doi:10.1371/journal.pone.0211927)
Supplement: S2 Table — (DOCX) [file pone.0211927.s004.docx]

**Supporting Information**

**S2 Table: Antibody panel 1 for NK cell subsets.**

| **Laser** | **mAb Name** | **Conjugation** | **Clone** | **Company** |
| --- | --- | --- | --- | --- |
| Blue (488) | NKG2A CD159a | VioBright FITC | REA110 | Miltenyi |
| Yellow/Green (561) | KIR3DL2 CD158k | PE | 539304 | R&D |
|  | CD27 | PE-CF594 | M-T271 | BD |
|  | KIR2DL2/DL3 CD158b | PE-Cy5.5 | GL183 | Beckman Coulter |
|  | KIR2DL1 CD158a | PE-Cy7 | EB6B | Beckman Coulter |
| Red (640) | KIR2DL5 CD158f | APC | UP-R1 | Miltenyi |
|  | KIR3DL1 CD158e | Alexa Flour 700 | DX9 | BioLegend |
| Violet (405) | CD3 | BV421 | UCHT1 | BD |
|  | CD14 | V500 | MP9 | BD |
|  | CD19 | V500 | HIB19 | BD |
|  | Live/Dead aqua | V500 |  | Invitrogen |
|  | CD56 | BV605 | HCD56 | BioLegend |
|  | CD16 | BV711 | 3G8 | BD |

Vitally frozen PBMCs from all patients were thawed, washed and resuspended in cold PBS supplemented with 2% FBS and 1mM EDTA. Antibody stainings were performed by incubating the cells with monoclonal antibodies at 4°C for 30 min in the dark. The labeled cells were then washed twice with PBS containing 2% FBS, 1mM EDTA prior to data acquisition.
