# Supplementary material for: Infectious complications and NK cell depletion following daratumumab treatment of Multiple Myeloma
Source: PLoS One. 2019 Feb 13;14(2):e0211927. doi: 10.1371/journal.pone.0211927 (PMC6374018; doi:10.1371/journal.pone.0211927)
Supplement: S5 Table — (DOCX) [file pone.0211927.s007.docx]

**Supporting Information**

**S5 Table: Antibody panel 4 for NK cell subsets.**

| **Laser** | **mAb Name** | **Conjugation** | **Clone** | **Company** |
| --- | --- | --- | --- | --- |
| Blue (488) | 2B4 CD244 | PerCP-Cy5.5 | C1.7 | Biolegend |
|  | Siglec9 CD329 | FITC | E10-286 | BD |
| Yellow/Green (561) | NTBA CD352 | PE | NT-7 | BioLegend |
|  | CD57 | PC-CF594 | NK-1 | BD |
|  | Siglec7 CD328 | PE-Vio770 | REA214 | Miltenyi |
| Red (640) | NKp44 CD336 | Alexa Flour 647 | p44-8.1 | BD |
|  | CD11b | Alexa Flour 700 | 4171579 | BD |
| Violet (405) | CD3 | BV421 | UCHT1 | BD |
|  | CD14 | V500 | MP9 | BD |
|  | CD19 | V500 | HIB19 | BD |
|  | Live/Dead aqua | V500 |  | Invitrogen |
|  | CD56 | BV605 | HCD56 | BioLegend |
|  | CD16 | BV711 | 3G8 | BD |

Vitally frozen PBMCs from all patients were thawed, washed and resuspended in cold PBS supplemented with 2% FBS and 1mM EDTA. Antibody stainings were performed by incubating the cells with monoclonal antibodies at 4°C for 30 min in the dark. The labeled cells were then washed twice with PBS containing 2% FBS, 1mM EDTA prior to data acquisition.
